# Spatiotemporal transformations for gaze control

**DOI:** 10.14814/phy2.14533

**Published:** 2020-08-18

**Authors:** Amirsaman Sajad, Morteza Sadeh, John Douglas Crawford

**Affiliations:** ^1^ Centre for Vision Research York University Toronto ON Canada; ^2^ Psychology Department Vanderbilt University Nashville TN USA; ^3^ Department of Neurosurgery University of Illinois at Chicago Chicago IL USA; ^4^ Vision: Science to Applications Program (VISTA) Neuroscience Graduate Diploma Program Departments of Psychology, Biology, Kinesiology & Health Sciences York University Toronto ON Canada

## Abstract

Sensorimotor transformations require spatiotemporal coordination of signals, that is, through both time and space. For example, the gaze control system employs signals that are time‐locked to various sensorimotor events, but the spatial content of these signals is difficult to assess during ordinary gaze shifts. In this review, we describe the various models and methods that have been devised to test this question, and their limitations. We then describe a new method that can (a) simultaneously test between *all* of these models during natural, head‐unrestrained conditions, and (b) track the evolving spatial continuum from target (T) to future gaze coding (G, including errors) through time. We then summarize some applications of this technique, comparing spatiotemporal coding in the primate frontal eye field (FEF) and superior colliculus (SC). The results confirm that these areas preferentially encode eye‐centered, effector‐independent parameters, and show—for the first time in ordinary gaze shifts—a spatial transformation between visual and motor responses from T to G coding. We introduce a new set of spatial models (T‐G continuum) that revealed task‐dependent timing of this transformation: progressive during a memory delay between vision and action, and almost immediate without such a delay. We synthesize the results from our studies and supplement it with previous knowledge of anatomy and physiology to propose a conceptual model where cumulative transformation noise is realized as inaccuracies in gaze behavior. We conclude that the spatiotemporal transformation for gaze is both local (observed within and across neurons in a given area) and distributed (with common signals shared across remote but interconnected structures).

## Graphical abstract

In this Review we describe challenges the literature has faced to study spatial transformations in the visuomotor pathway required for orienting to visual stimuli during natural head‐unrestrained gaze behavior. We summarize our relatively new method developed to address this question and review our recent studies that apply it to neural activity in two key structures in gaze control, the frontal eye field and superior colliculus. We synthesize these results and supplement it with the previous literature to propose a new conceptual model where cumulative transformation noise, within local and distributed circuits, is realized as inaccuracies in gaze behavior.

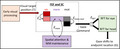

## 1 INTRODUCTION

A central question in sensorimotor neuroscience concerns what sequence of events takes place in order to transform vision into voluntary action (Bremner & Andersen, 2014; Bruce & Goldberg, 1985; Crawford, Henriques, & Medendorp, 2011; DeCharms & Zador, 2000; Flanders, Tillery, & Soechting, 1992; Gallivan & Culham, 2015; Gnadt, Bracewell, & Andersen, 1991; Goodale, 2011; Helmbrecht, Dal Maschio, Donovan, Koutsouli, & Baier, 2018; Optican, 2005; Pouget & Snyder, 2000; Robinson, 1973; Schall, 2019; Schall & Thompson, 1999; Sparks, 1986, 2002; Westendorff, Klaes, & Gail, 2010). One of the best studied experimental models in sensorimotor neuroscience is the gaze control system, which serves to orient the fovea toward visual stimuli. A gaze shift to a visual stimulus requires the appropriate movements of the eyes (and often the head) in space and time. Therefore, sensorimotor transformation is as much of a spatial problem as it is a temporal problem (Andersen, Snyder, Li, & Stricanne, 1993; Crawford et al., 2011; Franklin, Reichenbach, Franklin, & Diedrichsen, 2016; Heitz, 2014; Optican, 2005; Snyder, 2000). Surprisingly, the spatiotemporal transformations for ordinary gaze shifts (made directly or after a short delay toward a visual stimulus) have only recently been demonstrated.

Macaques have proven to be useful experimental models for studying gaze control circuitry due to the anatomical and functional similarities with the human system (Kaas, 2004; Passingham, 2009). Many neurons in the primate gaze system exhibit elevated discharge rate in response to a visual stimulus (visual response) and/or around the time of movement (motor/movement response; Bruce & Goldberg, 1985; Goldberg, Colby, & Duhamel, 1990; Hikosaka & Wurtz, 1983; Mays & Sparks, 1980; Mohler & Wurtz, 1976; Schall, 2015; Schlag‐Rey & Schlag, 1984). However, the spatial mapping between these temporal codes is not trivial. Numerous modeling and experimental studies have attempted to address this question (e.g. Basso & May, 2017; Cohen & Andersen, 2002; Crawford et al., 2011; Funahashi, Takeda, & Watanabe, 2004; Fuster, 2001; Gandhi & Katnani, 2011; Sato & Schall, 2003; Snyder, 2000; Sparks, 2002; Sparks & Mays, 1990). As we shall see, each of these approaches have provided important advances in understanding spatial coding for gaze control, and each has its limitations. Most importantly, traditional methodologies did not allow one to simultaneously test all spatial models, or track their progress through time. So much of what we believe about ordinary gaze transformations relies on inferences integrated from more complex laboratory paradigms.

The goals of this review are to (a) summarize a relatively new method to identify spatiotemporal codes in the brain, (b) describe the application of this method in two important oculomotor structures—the frontal eye field (FEF) and superior colliculus (SC)—during fairly ordinary head‐unrestrained gaze shifts, (c) use this as an opportunity to directly compare the neurophysiology of these two structures, and (d) contextualize these new results with respect to the classic oculomotor literature. The novelty of our approach is the use of a sophisticated computational analysis method that is able to simultaneously test between all of the known, as well as novel, spatial models in these structures through different task events (Keith, DeSouza, Yan, Wang, & Crawford, 2009; Sajad et al., 2015; Sajad, Sadeh, Yan, Wang, & Crawford, 2016). As we shall see, similar spatiotemporal transformations occur in both structures at the level of within and between neurons, suggesting that they occur at the level of shared, distributed signals rather than specific brain structures. First, we will provide some general background and review of the SC and FEF, of the spatial models that have been proposed, and the ways these have been tested.

## 2 OVERVIEW OF SC AND FEF ANATOMY AND ROLES IN GAZE CONTROL

**FIGURE 1:**
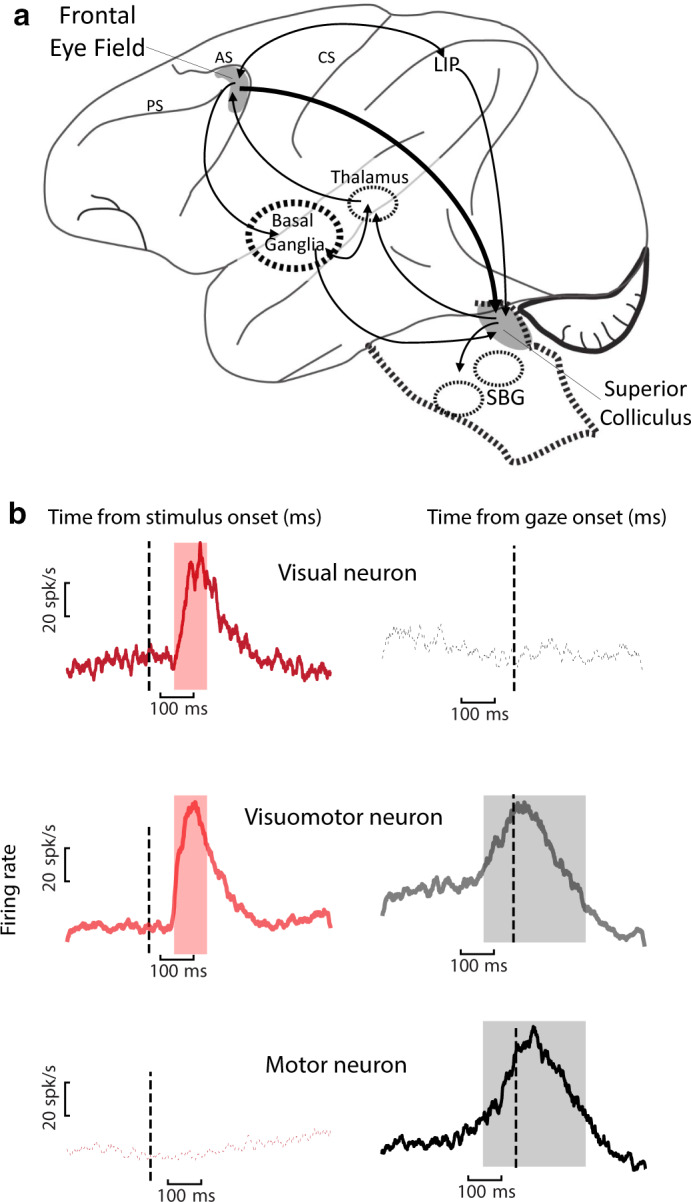
Schematic of key areas in dorsal visual pathway and representative visuomotor signals. (a) FEF and SC (shaded in gray) are shown in relation to interconnected structures. AC: arcuate sulcus, PC: Principal sulcus, CS: Central sulcus, SBG: Saccade Burst Generator. (b) Target‐ and gaze movement‐ aligned population responses of three general classes of neurons: Visual, Visuomotor, and Motor neurons in FEF (data from Sajad et al., 2015). Similar response profiles observed in SC (Sadeh et al., 2015). For the results reviewed in this manuscript, FEF and SC visual responses were sampled from 80 to 180 ms (pink shade) and 60 to 160 ms (not shown) following target presentation, respectively. FEF and SC motor responses included the bulk of the motor burst for each neuron (gray shade indicates mean FEF motor response). For SC, this window was fixed from −50 to +50 ms from gaze onset (not shown)

In macaques, the FEF is a cortical structure located at the bank of the arcuate sulcus, with large pyramidal neurons in layer 5, characteristic of cortical motor structures (Stanton, Deng, Goldberg, & McMullen, 1989; reviewed by Schall et al., 2017), whereas the SC is a multilayered subcortical structure located on the roof of midbrain (Mohler & Wurtz, 1976; reviewed by May, 2006). These two structures are intimately connected (Figure 1a): the FEF sends projections to the SC directly (Künzle, Akert, & Wurtz, 1976; Stanton, Goldberg, & Bruce, 1988a), and via the basal ganglia (Astruc, 1971; Hikosaka & Wurtz, 1983; Stanton, Goldberg, & Bruce, 1988b). The SC sends projections back to the FEF via the dorsomedial thalamus (Benevento & Fallon, 1975; Barbas & Mesulam, 1981; Goldman‐Rakic & Porrino, 1985; Lynch, Hoover, & Strick, 1994; Figure 1a). The SC and (to a lesser extent) the FEF project directly to the brainstem and spinal cord burst generators that innervate motoneurons for eye and head motion (Castiglioi, Gallaway, & Coulter, 1978; Harting, 1977; Huerta, Krubitzer, & Kaas, 1986; Isa & Sasaki, 2002; Kawamura, Brodal, & Hoddevik, 1974; Segraves, 1992; Stanton et al., 1988a). The causal role of the SC and FEF in gaze shift production is well established through various microstimulation (Bruce, Goldberg, Bushnell, & Stanton, 1985; Klier, Wang, & Crawford, 2001; Monteon, Constantin, Wang, Martinez‐Trujillo, & Crawford, 2010; Paré, Crommelinck, & Guitton, 1994), lesion (Schiller, Sandell, & Maunsell, 1987), and inactivation studies (Bollimunta, Bogadhi, & Krauzlis, 2018; Dias, Kiesau, & Segraves, 1995; Hanes & Wurtz, 2001; Hikosaka & Wurtz, 1985; McPeek & Keller, 2004). Both SC and FEF also receive direct visual input from the thalamus and visual cortex (Kaas & Huerta, 1988; Lynch et al., 1994; Schall, Morel, King, & Bullier, 1995). The superficial layer of the SC also receives direct visual input from the retina (Perry & Cowey, 1984).

Both the FEF and SC can exhibit visual and motor responses (Bruce & Goldberg, 1985; Hanes & Schall, 1996; Mohler & Wurtz, 1976; Munoz & Wurtz, 1995; Paré & Hanes, 2003; Schiller, 1984). Neurons in these structures are often classified according to their temporal responses (Figure 1b): Visual neurons exhibit visual response, Motor (or movement) neurons exhibit motor response, and Visuomotor (or Visuomovement) neurons exhibit both response types (Bruce & Goldberg, 1985; Wurtz & Albano, 1980; but see Lowe & Schall, 2018). Visual and motor responses in the FEF and SC are often spatially selective for a restricted patch of space called a “response field” (or “receptive field” for the visual response; Bruce & Goldberg, 1985; Mohler, Goldberg, & Wurtz, 1973; Mohler & Wurtz, 1976; Sparks, 1988). FEF and SC response fields are often tuned for the contralateral visual field, and SC receptive fields show anatomic topographic organization.

## 3 SPATIAL MODELS FOR GAZE

As noted in the introduction, just because a neural event coincides temporally with an externally observable event (i.e. visual stimulus or saccade onset), it does not mean that one can assume which spatial variable is encoded. This is particularly true of motor‐locked signals, which may (or may not) have undergone considerable processing after the initial sensory input. At the input level for gaze saccades, light from visual stimuli hit the photoreceptors on the retina. Because the retina is fixed on the eye, we can say that the retina encodes visual stimuli in an eye‐centered frame where the fovea is the origin and positions can be defined by vectors projecting outwards along the spherical retina (Demb & Singer, 2015). Ultimately, the gaze system uses this to evoke patterns of muscle contractions to move the eye (rotation in head) and head (rotation on body) toward the stimulus. What remains unclear is how eye‐centered stimulus representations are transformed into muscle coordinates. Despite decades of work, there is still no consensus on the sequence of spatial transformations in the gaze system. Here, we briefly review some of the alternatives that have been proposed, and ways they have been experimentally tested.

### 3.1 Canonical models in gaze control

**FIGURE 2:**
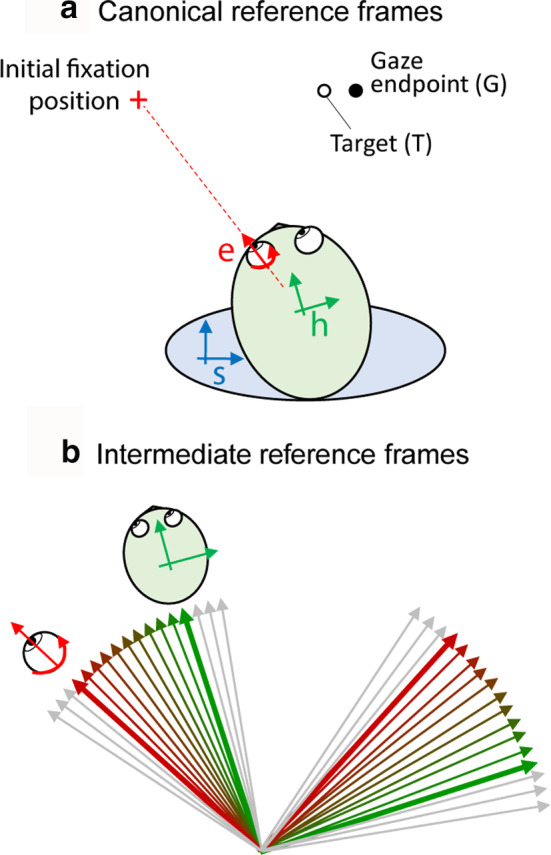
Spatial models in gaze control. (a) The location of the peripheral visual target (T) and the eventual location of the gaze shift (G) relative to egocentric reference eye (e), head (h), and body/space (s) reference frames at the time of fixation on the red cross. The spatial difference between T and G reflects inaccuracy in gaze behavior. (b) Intermediate eye‐head reference frames obtained by the linear combination of eye‐reference frame (red) and head‐reference frame (green) ranging from a more eye‐centered frame to a more head‐centered frame (color shade). Adapted from Sajad et al., (2015)

To characterize spatial processing in the brain, it is important to ask two questions: (a) what spatial parameter is encoded? and (b) what is the reference frame used to encode that parameter? (e.g. Soechting & Flanders, 1992). In the head‐unrestrained gaze control system, one might expect to encode spatial parameters, such as the visual target (T; e.g. Optican, 2005; Steenrod, Phillips, & Goldberg, 2012), eye motion (E), head motion (H), or their combination: gaze motion (G; e.g. Chen, 2006; Cowie & Robinson, 1994; Freedman & Sparks, 1997; Gandhi & Katnani, 2011). Spatial parameters related to these motions might be encoded either as displacement vectors relative to initial position (dE, dH, and dG) or final positions irrespective of initial position (Crawford & Guitton, 1997; Daemi & Crawford, 2015; Kardamakis & Moschovakis, 2009). Finally, each of these parameters might be encoded relative to various egocentric frames of references, including the eye (Te, Ge, Ee, and He), the head (Th, Gh, Eh, and Hh), or the body/space (body and space frames are indissociable when body does not move) (Ts, Gs, Es, and Hs; see Figure 2a; Boussaoud & Bremmer, 1999; Colby, 1998; Crawford et al., 2011; Lappi, 2016; Soechting & Flanders, 1992). Noteworthy that in experiments conducted in complete darkness, where the surrounding objects are not visible, egocentric frames are the focus because the possibility for object‐centered (i.e. allocentric) spatial representations is eliminated (but see Bharmauria, Sajad, Li, et al., 2020; Li et al., 2017). Many early conceptual models assumed that low‐level representations, such as Te, must be transformed into higher level frames, such as Th, to control movement (Andersen & Zipser, 1988; Soechting, Tillery, & Flanders, 1990), but more recent neural network studies have shown that this is not necessarily the case (Blohm, Keith, & Crawford, 2009; Pouget & Snyder, 2000; Smith & Crawford, 2005). Instead, the brain might make use of partial or intermediate reference frames, as discussed next.

### 3.2 Intermediate reference frames

While the canonical reference frames (above) describe the egocentric representations in gaze control, empirical data as well as computational studies have suggested evidence for reference frames that are intermediate (or hybrid) between these frames (Avillac, Deneve, Olivier, Pouget, & Duhamel, 2005; Jay & Sparks, 1984; Martinez‐Trujillo, Medendorp, Wang, & Crawford, 2004; Pouget & Snyder, 2000; Stricanne, Andersen, & Mazzoni, 1996). Quantitatively, intermediate reference frames are obtained from the linear combination of two canonical frames (Figure 2b). In Figure 2b, the eye frame (red) and head frame (green) and nine intermediate frames with different degrees of eye‐ and head‐centeredness are shown. Constructing these intermediate frames of reference allows one to test nuances that are missed when one forces the data into predefined categories (e.g. Caruso, Pages, Sommer, & Groh, 2018). Recently, we have extended the concept of intermediate spatial coding to the coding of spatial parameters *within* the same reference frame, a key topic which we will return to below. Other coding mechanisms—such as gaze‐dependent “gain fields” are likely important for implementing reference frame transformations (Andersen & Zipser, 1988; Blohm & Crawford, 2009; Salinas & Abbott, 2001; Smith & Crawford, 2005), but will not be the focus of the current review.

## 4 TRADITIONAL APPROACHES TO STUDYING SPATIAL ENCODING

While a spatially tuned stimulus‐locked response is most likely related to stimulus location, at least in the absence of recurrent feedback, further processing means that a movement‐locked response may either be related to the stimulus or the metrics of the imminent movement (Marino, Rodgers, Levy, & Munoz, 2008; Omrani, Kaufman, Hatsopoulos, & Cheney, 2017; Stanford & Sparks, 1994). Most behavioral paradigms that dissociate these locations suggest the latter: imminent movement (Everling, Dorris, Klein, & Munoz, 1999; Everling & Munoz, 2000; Funahashi, 2013; Zhang & Barash, 2000; but see Edelman & Goldberg, 2002; Frens & Van Opstal, 1997; Quessy, Quinet, & Freedman, 2010). Further, whereas most studies involve head‐restrained eye motion, in natural head‐unrestrained conditions, the same signal might encode eye motion, head motion, or the combination: gaze (Chen, 2006; Cullen, Galiana, & Sylvestre, 2000; Guitton, Munoz, & Galiana, 1990; Knight, 2012; Paré & Guitton, 1990; Sparks, Freedman, Chen, & Gandhi, 2001; Walton, Bechara, & Gandhi, 2007). Under such conditions, many of the models described in the previous section become impossible to disentangle. Below we will review the traditional approach to investigating spatial parameters and their respective reference frames in FEF and SC.

### 4.1 Differentiating spatial parameters

The simple geometry of the oculomotor system actually imposes a challenge for testing spatial parameters. Unlike the reach system (where the visual vector and hand movement vector do not align unless the hand starts moving from the location of the eyeball; e.g. Blohm & Crawford, 2007), in the saccadic system, sensory and motor parameters are highly correlated (Freedman & Sparks, 1997; Marino et al., 2008; Smith & Crawford, 2005; Snyder, 2000). One way to overcome this challenge is to study random variations between these parameters (Bremmer, Kaminiarz, Klingenhoefer, & Churan, 2016; Keith et al., 2009; Platt & Glimcher, 1998; Wimmer, Nykamp, Constantinidis, & Compte, 2014), but neurophysiology techniques that rely on averaging often wash these out. For example, the variable scatter of gaze endpoint around target in many cases averages to zero, making it impossible to know if the activity of neurons is best described by target location or the gaze endpoint position. To overcome this limitation, experimenters have used clever paradigms that spatially dissociate the location of visual stimulus from the gaze target. Some have used motor adaptation paradigms in which after a training period, the motor system generates a movement that is spatially distinct from that of the visual stimulus (Edelman & Goldberg, 2002; Frens & Van Opstal, 1997; Quessy et al., 2010; Takeichi, Kaneko, & Fuchs, 2007). Others have used experimental tasks that require a deliberate (rule‐based) calculation of the gaze target to another location defined by (but different from) the visual stimulus (Everling et al., 1999; Everling & Munoz, 2000; Sato & Schall, 2003; Watanabe & Funahashi, 2007; Zhang & Barash, 2000). The most popular example of such tasks is the antisaccade task in which the subject is required to elicit an eye movement opposite to the direction of the target (Munoz & Everling, 2004). However, such transformations appear to be driven by top‐down feedback, propagating “backwards” from frontal to parietal to occipital cortex (Blohm et al., 2019; Paneri & Gregoriou, 2017). These techniques are thus valuable for understanding how the brain implements rule‐based, top‐down transformations, but they do not trivially map onto the standard bottom‐up sensorimotor transformations (Hawkins, Sayegh, Yan, Crawford, & Sergio, 2013; Jamadar, Johnson, Clough, Egan, & Fielding, 2015; Johnston, DeSouza, & Everling, 2009).

### 4.2 Differentiating reference frames

**FIGURE 3:**
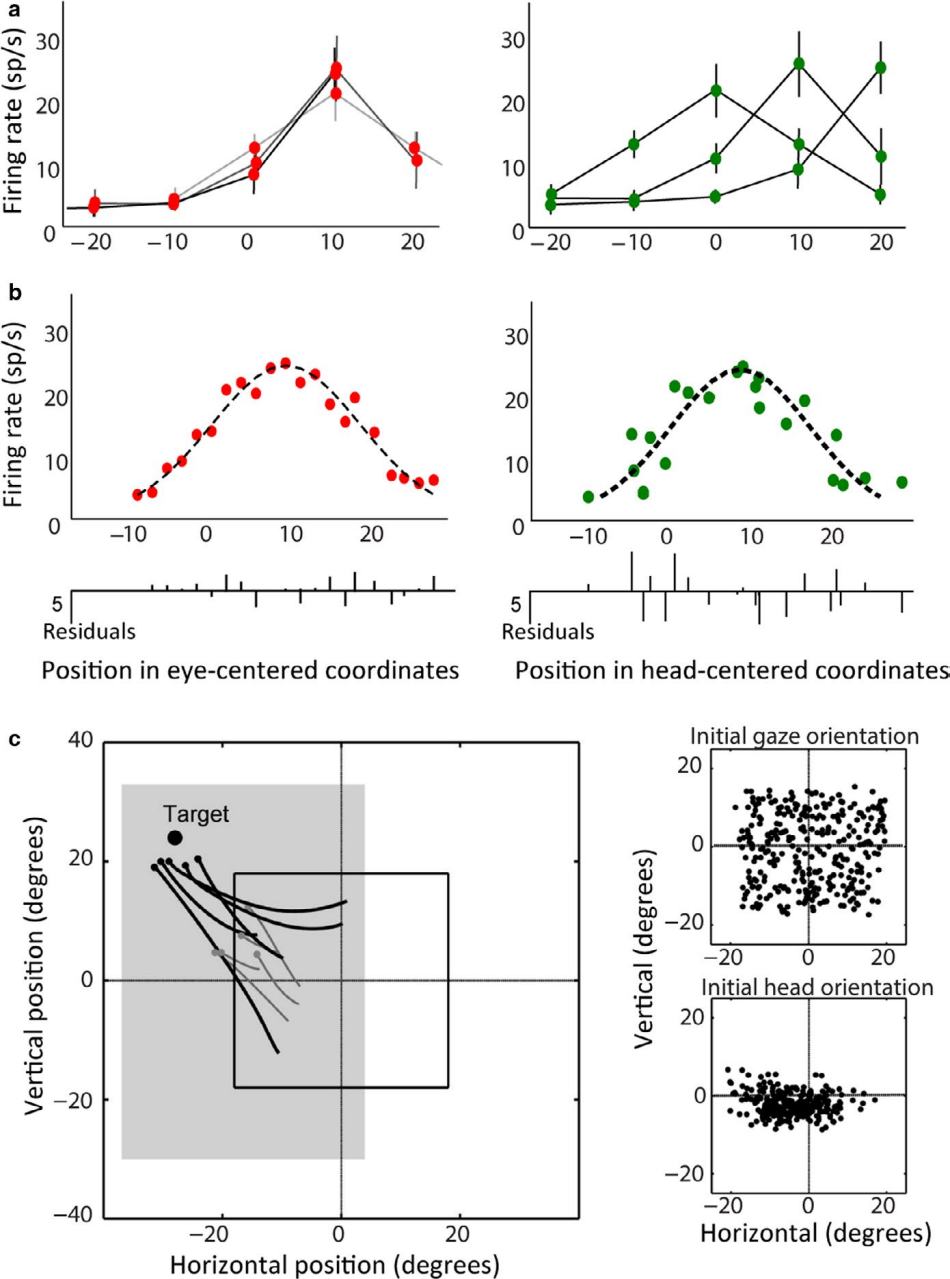
Classic and new methods of neural reference frame analysis for gaze control. (a) Response field plots obtained using the traditional approach to identify reference frames for an example neuron (e.g. Cohen & Andersen, 2002). Many trials are sampled while the head faces the front and the eye initial orientation varies between discrete positions. The shift in response field based on varying initial positions is assessed. The profile of the response field is conserved when plotted relative to the initial position of the eye (left), but shifts when plotted based on the initial position of the head (right), hence eye‐centered. (b) Response field plots reduced to one‐dimension, illustrating the logic for the statistical modeling method developed by Keith et al., (2009). Response fields were plotted by placing firing rate data over positions in space as defined by the tested model and the quality of the fit was assessed by measuring PRESS residuals obtained from a “remove one–fit–replace” approach (bottom panel shows residuals for all data points to a single fit). The response field is more spatially organized when plotted relative to initial eye orientation (left) compared to initial head orientation (right) as the data points (dots) fall closer to the nonparametric fit (dashed line, here looks Gaussian), hence eye‐centered. (c) In head‐unrestrained conditions, the dissociation of spatial parameters in gaze behavior were achieved by variability in eye‐head behavior. Gaze (black) and head (gray) movement trajectories to a single target (large circle) for five trials in the memory‐guided gaze task are shown (left panel). Gaze and head endpoint positions (small circles) fall at variable positions for the same target. Initial gaze position was randomly varied within a central square (black square) to increase variability in starting gaze orientation (upper‐right panel) and head orientation (lower‐right panel). This variability allowed for a differentiation between eye‐, head‐, and space‐ (or body) frames of reference. Adapted from Sajad et al. (2015)

Classically, reference frames under head‐immobilized conditions are investigated by systematically switching the initial eye orientation between several discrete positions (Figure 3a). Because the head is stationary relative to the body, the head and body/space frames remain in register. If the neural response shows systematic changes as a function of position relative to one effector but not the other, then the neuron's response is in the reference frame fixed to that effector (Avillac et al., 2005; Caruso et al., 2018; Cohen & Andersen, 2002; Jay & Sparks, 1984; Russo & Bruce, 1994). To investigate intermediate frames of reference, some studies use the quantitative definition to explicitly test for these frames (e.g. Avillac et al., 2005; Jay & Sparks, 1984; Figure 2b). However, these techniques do not separate the head and body frames, and require repetition and averaging that are difficult to replicate under natural head‐unrestrained conditions (Keith et al., 2009).

An overarching theme is that, while various ingenious methods have been used to test spatial models for vision and gaze control, they each have their own limitations, testing only parts of the question. In the following section, we describe a method that allows one to test all such models simultaneously during natural, head‐unrestrained conditions.

## 5 A MODEL‐FITTING APPROACH DEVELOPED FOR IDENTIFYING NEURONAL SPATIAL CODES

The following section describes an analytic approach that was developed to test between multiple models of spatial coding in neural activity during head‐unrestrained gaze behavior. The method can be viewed as complementary to decoding approaches, where machine learning algorithms are used to derive specified information from neural data (Bremmer et al., 2016; Leavitt, Pieper, Sachs, & Martinez‐Trujillo, 2017; Glaser et al., 2017; Pruszynski & Zylberberg, 2019). The latter approach tests for *implicit* population codes, whereas the current method tests for explicit coding, at the level of both single units and neural populations. To do this in the presence of complex and “sloppy” head‐unrestrained behavior, several technical challenges had to be overcome.

### 5.1 Challenges and benefits of head‐unrestrained gaze recordings

Head‐unrestrained experiments provide the potential benefits of allowing more natural gaze behavior, testing effector coding specificity (gaze vs. eye vs. head), and separating more frames of reference (eye vs. head vs. space/body). However, they also produce major analytic challenges. One is that correlative techniques are insufficient because gaze, eye, and head motion always correlate with each other. Another is that in the range of head‐unrestrained gaze motion, three‐dimensional (3‐D; horizontal, vertical, and torsional) measurements become important because torsional rotation of the eyes and head becomes more prominent, and linear operations on 2‐D gaze/eye/head signals (only horizontal and vertical) yield large errors related to noncommutativity (Tweed, Haslwanter, Happe, & Fetter, 1999). Likewise, this requires a 3‐D analysis to accurately compute positions, such as Te and Ge, which are positions in true retinal (i.e. eye‐centered) coordinates (Crawford et al., 2011). A third challenge is that even for the same gaze orientation, the relative orientations of eye and head can be highly variable (DeSouza et al., 2011; Freedman & Sparks, 1997). Consequently, the traditional approach for identifying the reference frames (Figure 3a) is difficult to replicate. On the other hand, as we shall see, these same problems can be turned into advantages (Figure 3b,c).

Figure 3c illustrates the aspects of gaze behavior that we have utilized to map SC and FEF response fields in several of our recent studies (Sadeh, Sajad, Wang, Yan, & Crawford, 2015, 2018, 2020; Sajad et al., 2015, 2016). Important for addressing the spatial code is the pattern of various spatial parameters during this task. The (largely self‐generated) variability in the behavior tends to separate spatial parameters. The animal's gaze end‐points form a scatter around a given target, separating T and G (Figure 3c, left panel). The animal itself uses different combinations of eye and head rotation (Figure 3c. top‐right panel; including torsion, not shown) to achieve a given gaze shift, separating different effectors. Likewise, the animal uses different combinations of initial eye and head position (Figure 3c, bottom‐right panel; including torsion, not shown), which separate out different frames of reference. To increase the separation between the frames of reference, we introduced an additional variability in the initial gaze positions. Now, all that is needed is some statistical method able to account for these variations and utilize them to fit various spatial models against neural activity.

### 5.2 New approach to studying spatial encoding using PRESS statistics

**FIGURE 4:**
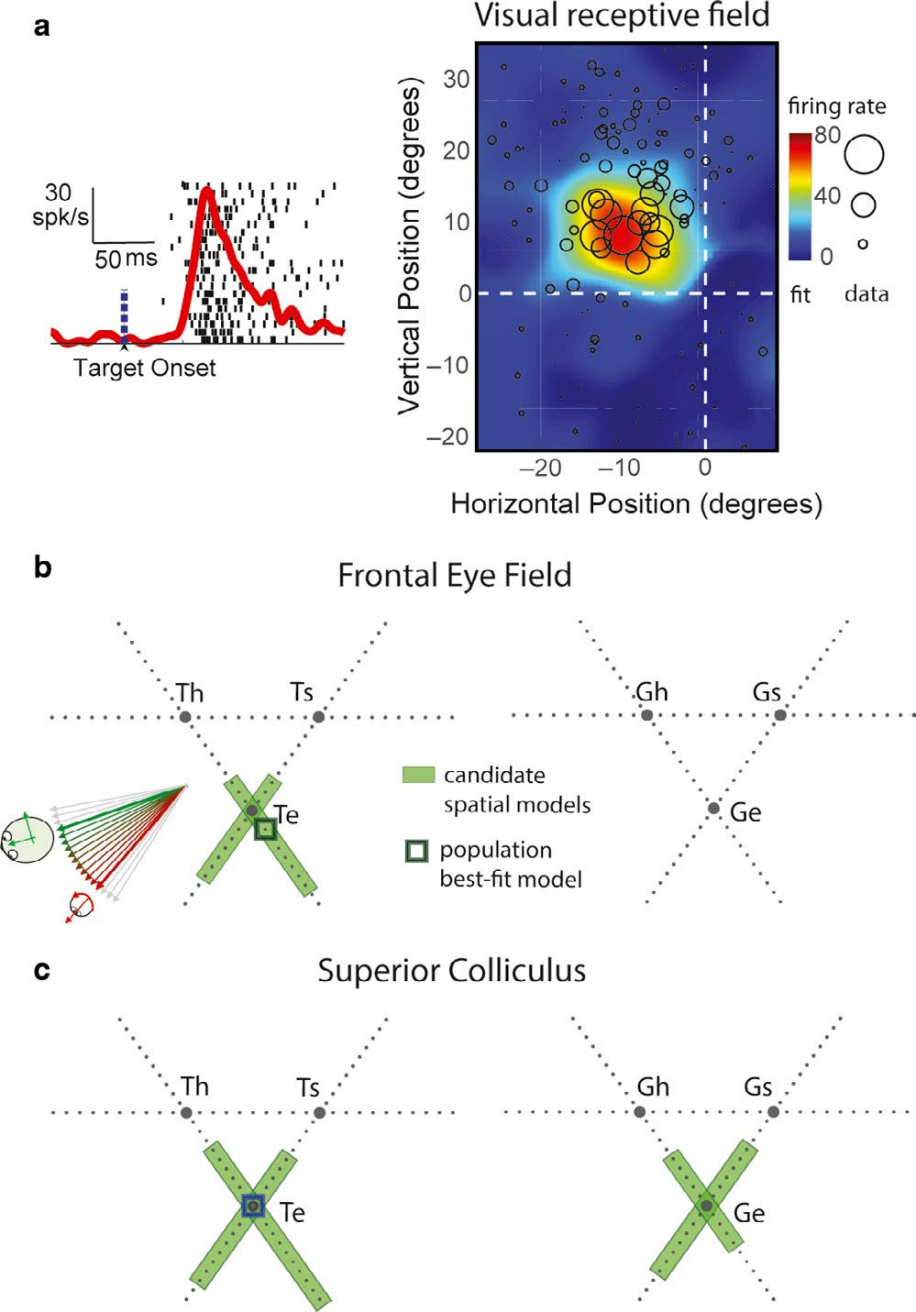
Spatial analysis of visual receptive fields in FEF and SC. (a) Raster and spike density function aligned on target onset (left) and the visual receptive field plot (right) of a representative visual response in FEF. Circles (radius: firing rate) represent data points for response field mapping. Activity was sampled from the 80–180 ms after target presentation (Figure 1b). Color‐map represents the nonparametric fit to the data. (b) Triangular plots represent intermediate models constructed from three pairs of canonical models: eye (e), head (h), and body/space (s) frames based on target location (left) and gaze endpoint (right). The continua between eye and head intermediate frames (Te‐Th, and Ge‐Gh) are also shown in Figure 2b. Green shade indicates intermediate spatial models that are not significantly eliminated. Black square indicates the population best‐fit model. (c) Similar conventions as (b) for superior colliculus. Green shades in (b) and (c) cluster around eye‐centered T (Te) and G (Ge) models. The population best‐fit (dark green square) was at intermediate spatial model at or close to Te for both FEF and SC. Adapted from Sajad et al., (2015) and Sadeh et al., (2015)

**FIGURE 5:**
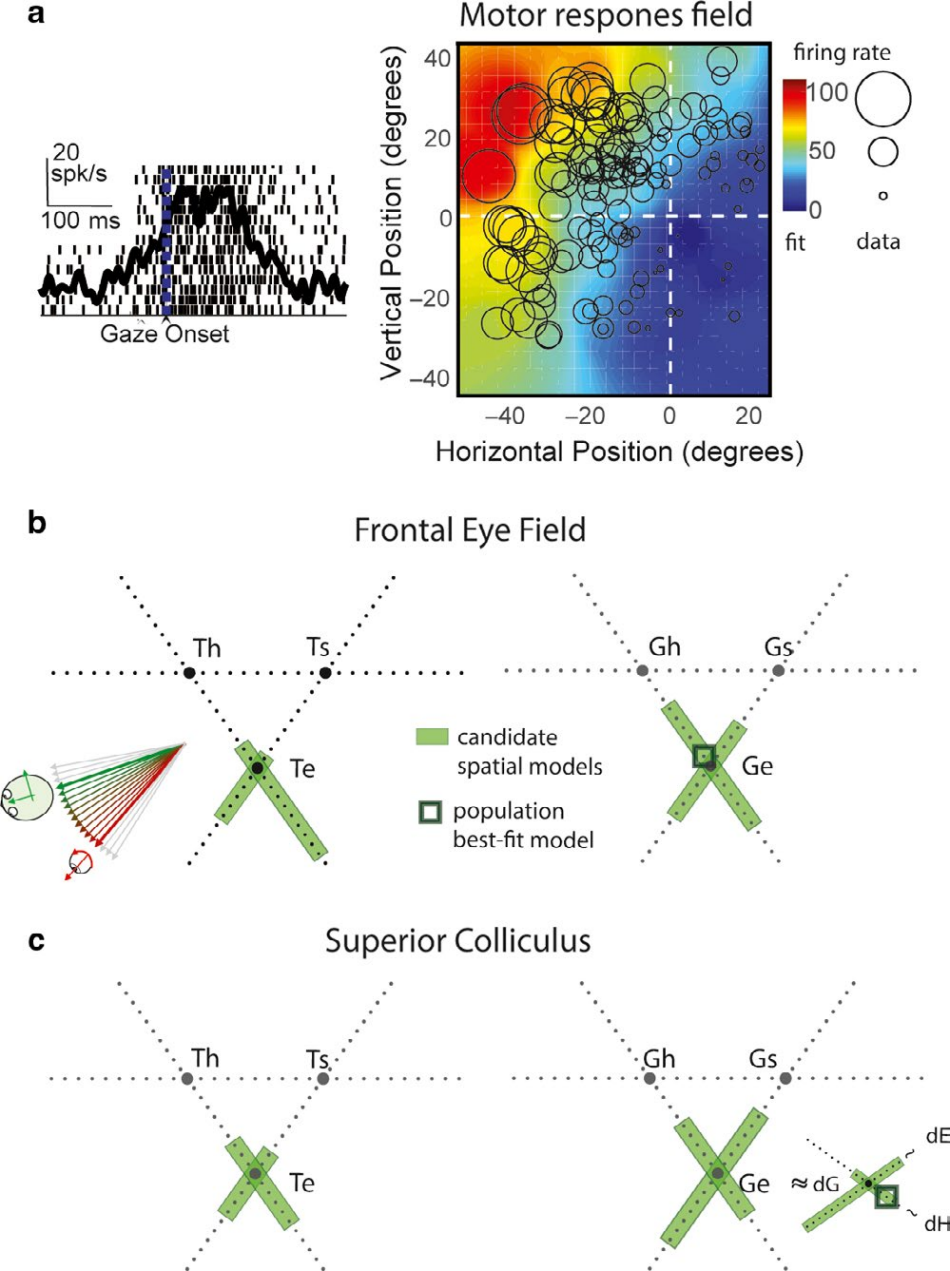
Spatial analysis of motor response fields in FEF and SC. (a) Raster and spike density function aligned on gaze onset (left) and the motor response field plot (right) of a representative FEF motor response. Similar conventions as Figure 4a. (b and c) Spatial analysis of motor response fields of FEF (b) and SC (c) neurons. Similar conventions as Figure 4b,c are used. Motor response was sampled from the entire motor response (Figure 1b). Notice that noneliminated intermediate models (green shades) cluster around eye‐centered T (Te) and G (Ge) models. The population best‐fit for FEF motor activity was an intermediate spatial model close to Ge, and for SC motor activity was an intermediate model close to dG (gaze displacement), which is geometrically very similar to Ge. Adapted from Sajad et al., (2015) and Sadeh et al., (2015)

To overcome the above challenges, Keith et al., (2009) introduced a method, which takes advantage of the property that neurons have spatially organized response fields. To identify the spatial parameter and reference frame that best describe variations in the neuron response, they exploited the natural variability in behavior described above. Figure 3b depicts the logic for this approach. Neural activity is plotted against each set of spatial parameters derived from the behavioral data. Spatial models were constructed by nonparametric fits through the distribution of data. Then, the quality of the fit for each model is quantified using Predicted Residual Error Sum of Squares (PRESS) statistics which is a form of cross‐validation used in regression analysis (Keith et al., 2009). In other words, for each data point, the residual is calculated relative to a fit to all the other data points, excluding the point in question. The spatial model that yields the lowest PRESS residuals (i.e. the best‐fit) is assumed to characterize the spatial parameter the neuron encodes, and models that yield significantly larger residuals (at the single neuron or population level) can be eliminated from consideration. This method can also be adapted to fitting intermediate models. For example, one can construct models based on points between and beyond the Te and Th (Figure 2b) and determine which weighting yields the lowest overall residuals (e.g. Figures 4b,c and 5b,c). As shown in these figures, this method is easiest to visualize with 2‐D response fields, but in principle, it can be applied to neurons that encode any spatially variable behavior in any multidimensional coordinate system. In the following sections, we review the use of these methods to describe response fields and spatial coding, for the first time directly comparing our results from the FEF and SC.

## 6 VISUAL RECEPTIVE FIELDS

The current viewpoint is that the visual response in both the FEF and SC can be characterized by a salience or priority map of space (Fernandes, Stevenson, Phillips, Segraves, & Kording, 2013; Krauzlis, Lovejoy, & Zénon, 2013; Thompson & Bichot, 2005; White et al., 2017), but what spatial parameter and reference frame code does this map employ? (note that this is not the same as “retinotopy”, which is the way these signals are anatomically distributed). Most previous studies that have explicitly tested for reference frames suggest that SC and FEF visual responses encode the visual stimulus location fixed in retinal coordinates (e.g. Bruce & Goldberg, 1985; Cassanello & Ferrera, 2007; Lee & Groh, 2012; Schiller & Stryker, 1972; Snyder, 2000; but see Caruso et al., 2018), so this is a good place to test and confirm the new method described above.

DeSouza et al. (2011) were the first to investigate the reference frame of visuomotor responses in the SC in head‐unrestrained conditions using an early version of the method described above (DeSouza et al., 2011). They sampled visuomotor responses during visually guided gaze shifts and found that, overall, variations in combined SC visuomotor responses were best described by target location (and not final position of gaze) in eye‐centered coordinates. However, visual responses were not clearly separated from motor responses in that experiment.

More recently, we gathered data from both the SC and FEF during an oculomotor delayed memory‐guided task, which temporally separates the visual and motor responses intervened by a short memory delay (Sadeh et al., 2015; Sajad et al., 2015). We found that across the complete set of spatial models tested (see Section 2), perhaps not surprisingly, those related to the movement of the eyes (in the head) and the head (on the body) were eliminated. Indeed, the vast majority of visually responsive neurons in both FEF and SC had response fields that exhibited the highest spatial organization (and lowest residuals of fit) when they were plotted based on target position in eye‐centered coordinates (Te; Figure 4a). At the population level (all neurons with visual responses), these fits were significantly better than any other model, and sometimes the preference for eye‐centered coding was statistically significant even at the level of individual neurons.

However, it might be argued that by restricting our fits to canonical models, especially at the population level, one might miss either systematic or variable shifts of individual neuron coding distributions along intermediate frames, away from the canonical models. Therefore, we did a comprehensive testing of intermediate reference frames constructed based on target and gaze endpoint positions (intermediate reference frames between each pair of reference frame, eye‐head, head‐space, and eye‐space). This analysis showed that although single FEF and SC visual neurons showed variable distributions along intermediate points between models, these distributions tended to mainly cluster around Te (Figure 4b,c). Based on these results, we concluded that the visual response in both FEF and SC encodes positions in eye‐centered coordinates.

## 7 MOTOR RESPONSE FIELDS

The nature of coding of the SC and FEF motor responses has been the subject of more debate than the visual response. Most visual‐motor dissociation tasks suggest that the motor response in FEF and SC encodes saccade direction (e.g. Everling et al., 1999; Everling & Munoz, 2000; Moon et al., 2007; Sato & Schall, 2003), but some have shown evidence for encoding sensory stimulus location (Edelman & Goldberg, 2002; Frens & Van Opstal, 1997; Quessy et al., 2010). Also as mentioned above, it is not known how results from these studies translate to ordinary saccades in which visual‐motor dissociations are absent, and the subject has to directly shift gaze toward the visual stimulus. There are also disagreements about the nature of the spatial code in FEF and SC related to eye‐head gaze behavior. Most head‐unrestrained studies have concluded that gaze (rather than eye or head) is the primary code (Freedman & Sparks, 1997; Guitton & Mandl, 1978; Klier et al., 2001; Knight & Fuchs, 2007; Monteon et al., 2010). Also studies in head‐restrained monkeys that recorded from neck muscle activity have drawn similar conclusions (Corneil, Olivier, & Munoz, 2002; Elsley, Nagy, Cushing, & Corneil, 2007). But some studies have shown evidence for independent eye and head movement coding in these structures (Bizzi & Schiller, 1970; Chen, 2006; Knight, 2012; Walton et al., 2007). Finally, the majority of reference frame studies suggest that an eye‐centered code predominates in FEF and SC (Bruce & Goldberg, 1985; Cassanello & Ferrera, 2007; Klier et al., 2001; Russo & Bruce, 1994; Schiller & Stryker, 1972; Snyder, 2000), but yet again, there are alternative views (Caruso et al., 2018). Some of the disagreements are due to differences in experimental conditions and assumptions about the behavior or neuronal spatial code. For example, if one assumes neurons encode a certain parameter (e.g. target position in many studies) without explicitly testing this, the traditional analysis method of reference frames could yield inaccurate conclusions especially if neurons encode other spatial parameters that show systematic variations relative to the assumed parameter.

We re‐examined this question by applying our model‐fitting approach to motor responses that accompanied head‐unrestrained gaze shifts, following the visual responses (described above) and memory delay (Sadeh et al., 2015; Sajad et al., 2015). We found that the motor response in both FEF and SC, similar to the visual response, showed a strong preference for eye‐centered models. Head‐centered and body/space‐centered models were significantly ruled out at the population level. Importantly, spatial models based on independent eye (in head) and head (in space) position and displacement were also significantly ruled out for both FEF and SC motor responses. Overall, Ge (and very similar model dG) gave the best fits, although Te was not eliminated.

Across the tested intermediate reference frames, for both FEF and SC, similar to the visual response, target and gaze position spatial models based on high degree of eye‐centeredness (but not head‐ and space‐centeredness) were preferred (Figure 5). However, unlike the visual response, the overall best‐fit model for motor response was a model closest to Ge (Figure 5b,c; or gaze displacement, dG, which is a very similar model to Ge; see Figure 5c), previously shown to be a better descriptor of SC motor output (Klier et al., 2001).

## 8 VISUOMOTOR TRANSFORMATIONS

**FIGURE 6:**
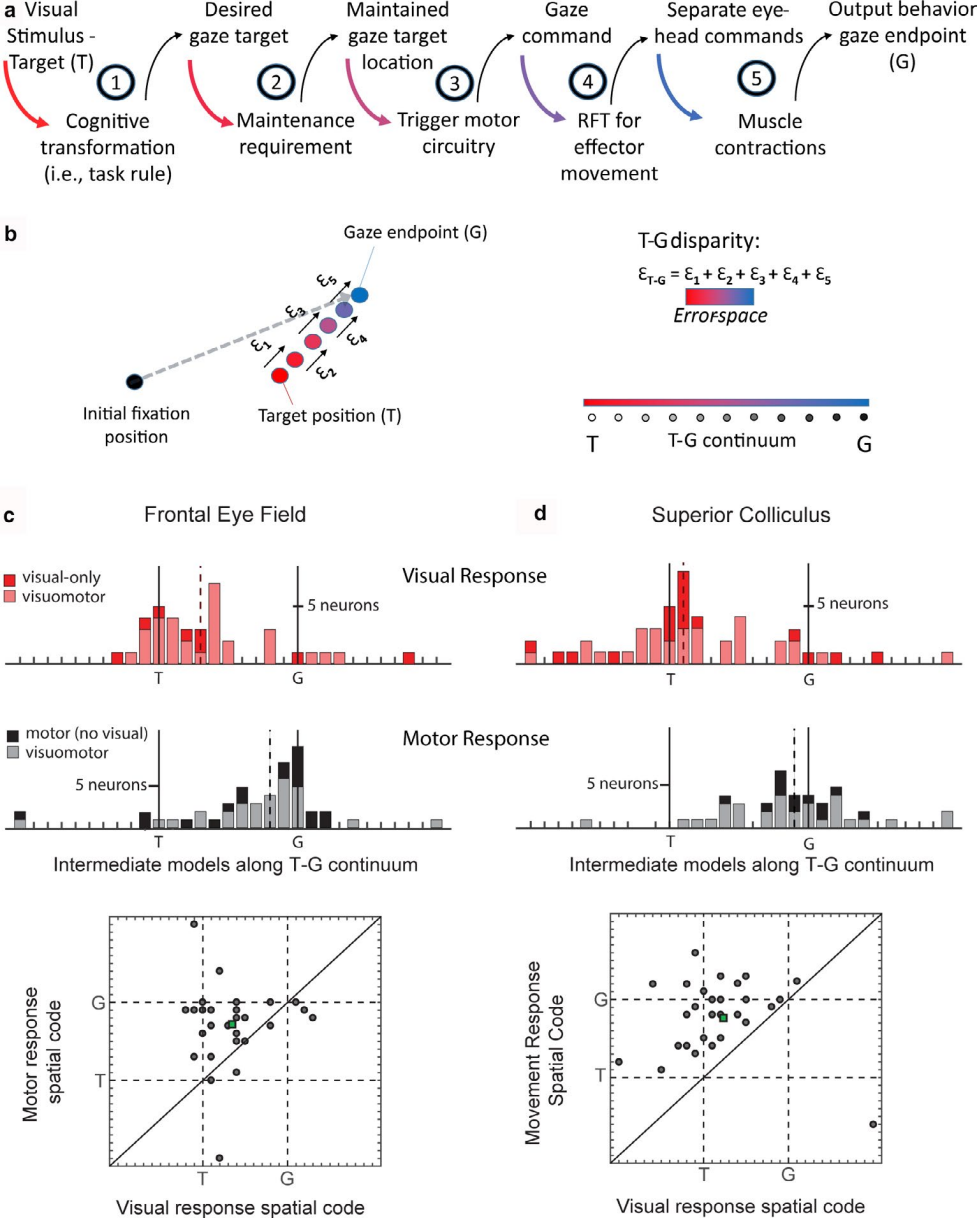
Visuomotor transformations in FEF and SC between visual and motor responses in memory‐guided gaze task. (a) Breakdown of stages in the transformation from target sensory information to output gaze behavior. (b) Red dot: location of the visual target (T); Each process can incrementally add to inaccuracies in spatial representation of target (Ɛ1‐5) resulting in inaccuracy in gaze behavior (gray dotted arrow: gaze vector; blue dot: gaze endpoint). We constructed the T‐G continuum by dividing the error‐space (i.e., T‐G disparity) into equal intervals. This allowed us to explicitly test whether neural activity prefers intermediary positions along this error‐space. Distribution of best‐fit model along the T‐G continuum for visually responsive neurons (c and d top panels) and motor‐responsive neurons (c and d, middle panels). FEF and SC visual responses were sampled as indicated in Figure 1b. Note: visuomotor neurons (pink, c and d top, and gray, c and d bottom) appear on both upper and lower panels. Scatter plots show the best‐fit model distribution of motor response (*y*‐axis) versus visual response (*x*‐axis) for individual Visuomotor neurons. Deviation from line of unity indicates change in spatial code along T‐G continuum between visual and motor response in Visuomotor neurons. Adapted from Sajad et al., (2015) and Sadeh et al., (2015)

Thus far, we have described visual and motor response field fits, without considering how the former is transformed into the latter. Visuomotor transformation potentially involves multiple computational stages, each of which can contribute to inaccuracies in gaze behavior (Alikhanian, Carvalho, & Blohm, 2015; Churchland, Afshar, & Shenoy, 2006; Faisal, Selen, & Wolpert, 2008; Gnadt et al., 1991; Ma, Husain, & Bays, 2014; Spaak, Watanabe, Funahashi, & Stokes, 2017; van Beers, 2007; van Bergen, Ma, Pratte, & Jehee, 2015; White, Sparks, & Stanford, 1994; Wimmer et al., 2014). Figure 6a shows a general breakdown of these stages: (a) visual target stimulus location (T) must be integrated with task rules to work out a desired gaze target (Miller & Cohen, 2001). Although we purposefully avoided this in our studies, in certain paradigms task rules are introduced to spatially dissociate stimulus location from the desired gaze location (Munoz & Everling, 2004). (b) Sometimes the gaze target needs to be maintained in working memory for a delayed response (Curtis, Rao, & D'Esposito, 2004; Gnadt et al., 1991). (c) This representation then needs to be relayed to the motor circuitry where the gaze command is generated (Chatham & Badre, 2015; Schall, Purcell, Heitz, Logan, & Palmeri, 2011). (d) This gaze command needs to be decomposed into separate effector commands to rotate the eye in head and the head on the body (Daemi & Crawford, 2015; Gandhi & Sparks, 2007; Guitton, 1992). (e) The separate eye and head movement commands then result in muscle contraction patterns that result in repositioning the gaze (G). Figure 6b shows that the noise in spatial representations associated with each stage (represented by *Ɛ*_stage_) can push the spatial code along the error‐space from T toward G, resulting in the overall inaccuracy in gaze behavior (i.e., T‐G disparity). Where along this sequence of information processing do FEF and SC visual and motor responses lie?

### 8.1 Introducing T‐G continuum—transformation of spatial code along the error‐space

To address the question posed in the last paragraph, we created a spatial continuum between Te and Ge, analogous to the idea of intermediate frames of reference, except that Te and Ge are both in the same eye‐centered frame of reference (Figure 6b). What separates these two parameters are variable inaccuracies in gaze behavior. We refer to this spatial continuum “T‐G continuum” as a set of spatial models spanning the *error‐space*. Accordingly, a change in spatial code from Te toward Ge (henceforth, we will refer to the eye‐centered codes Te and Ge as T and G for simplicity) reflects the incremental accumulation of inaccuracy in spatial representations along the visuomotor pathway, realized as variable errors in gaze behavior.

Figure 6c,d show the results of this analysis for FEF and SC visual (before a memory delay) and motor responses (after a memory delay). As one can see, visual responses clustered around T and motor responses clustered around G. Importantly, the shift from T to G was significant for both brain structures. This was also observed in plots of the motor versus visual T‐G continuum fits for individual Visuomotor neurons. Note that although these data were collected in head‐unrestrained conditions, these particular results would be expected to hold in head‐restrained conditions, because they do not depend on separation of effectors or frames.

Based on these observations, we concluded that the FEF (Sajad et al., 2015) and SC (Sadeh et al., 2015) are involved in the spatial visual‐to‐motor transformations for gaze shifts. Furthermore, they show that this happens both within and between neurons in both structures, suggesting a signal transformation that occurs at the cellular level but is distributed across brain structures.

### 8.2 Timing of the transformation within and between neurons in memory delay task

**FIGURE 7:**
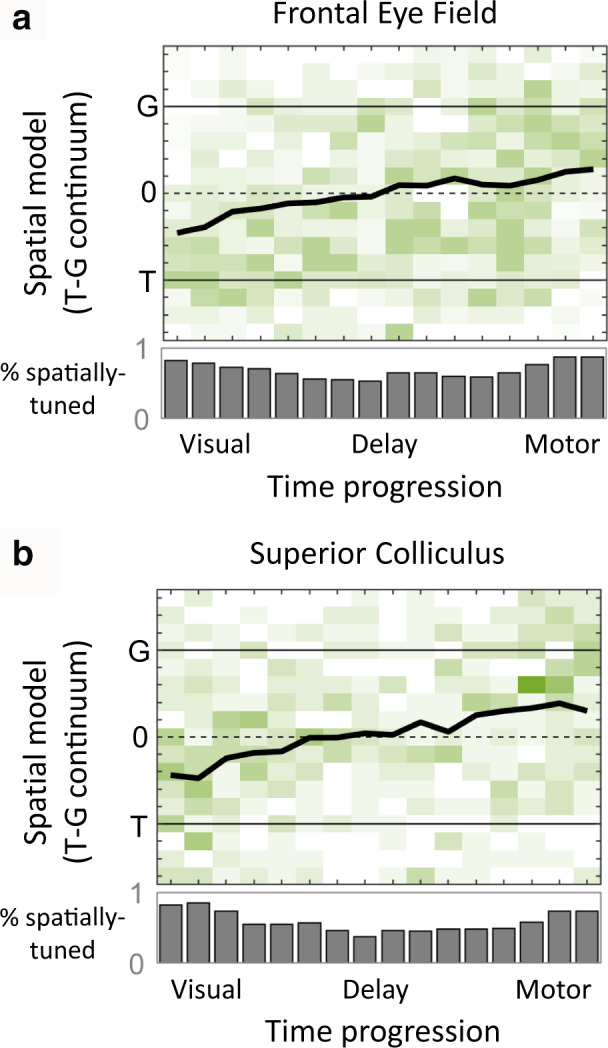
Temporal progression of spatial code during visual‐memory‐motor periods of the memory‐guided task. (a) The time course of T‐to‐G transition across all neurons in the FEF is shown for time intervals spanning visual response onset until saccade time. Green shades represent the best‐fit model for individual neurons. Black traces represent population mean of the best‐fit distribution. Gray histograms indicate the percentage of spatially tuned neurons at each time step. Adapted from Sajad et al., (2016). (b) Same analysis on SC neuronal responses

With the visual and motor responses in FEF and SC separated by a memory delay, does the transformation from T to G occur before, during, or after the memory delay? Furthermore, what is the differential contribution of different neuron types to this transformation? To address these questions, Sajad et al., (2016) examined the time course of the evolution of the spatial tuning along the T‐G continuum (i.e. error‐space) for FEF neurons by analyzing multiple time steps spanning an early visual period, the memory delay, and the motor response. We found that at the population level, the transition from T to G was characterized as monotonic and gradual through time during the entire visual‐memory‐motor intervals of the task (Figure 7a). A similar analysis of the SC neuronal data from Sadeh et al., 2015, done expressly for this article, revealed the same intermediate spatiotemporal transformation as the FEF (Figure 7b).

As described in more detail in the original paper (Sajad et al., 2016), further details emerged when we broke our FEF population down into four distinct subclasses. This revealed a number of fascinating details: Visual neurons encoded T, Visuomotor neurons showed an overall transition like the entire population with a visual code that was close to T but shifted toward G (Figure 7a), neurons with both delay and motor activities had a code that remained fixed between T and G, and neurons with motor‐only activity showed a significant further “jump” to G at the end. Sajad et al., (2016) interpreted this latter jump as evidence for a memory‐motor transformation within the FEF.

Overall, these results demonstrate that FEF and SC spatial codes evolve progressively along almost the entire T‐G range during a memory delay, and that different neuron types can contribute differently to a visual‐memory‐motor transformation, much like a relay team (Cohecelln, Pouget, Heitz, Woodman, & Schall, 2009; Heinzle, Hepp, & Martin, 2007; Lawrence, White, & Snyder, 2005; Markowitz, Curtis, & Pesaran, 2015; Merrikhi et al., 2017; Shin & Sommer, 2012; Spaak et al., 2017; Wurtz, Sommer, Paré, & Ferraina, 2001).

### 8.3 Rapid transformation during reactive gaze shifts

**FIGURE 8:**
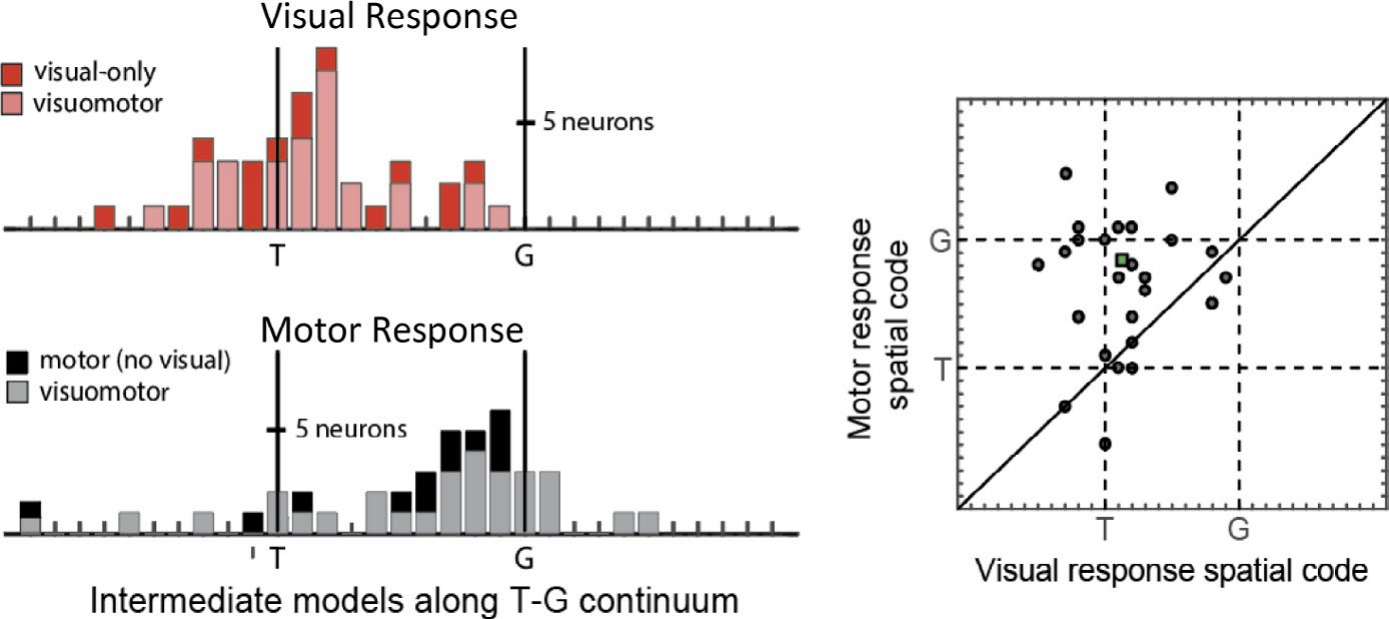
Visuomotor transformations between SC visual and motor response during visually guided (reactive) gaze task. Similar conventions as Figure 6d. Visual response was sampled from 60 to 160 ms relative to target onset, and motor response from −50 to +50 ms relative to gaze onset. Adapted from Sadeh et al., (2020)

Does the T‐to‐G transformation described in the previous sections depend on memory‐related processing? Conversely, can a similar transformation be demonstrated in simple saccades made directly to a target with no delay? To address these questions, Sadeh et al. (2020) recorded the activity of SC neurons during a direct visually guided gaze task (reactive task). As expected, gaze behavior was still inaccurate in this task albeit more accurate than the memory‐guided task, likely due to a lack of memory‐dependent processes vulnerable to noise (Figure 6a). This inaccuracy in behavior (i.e., disparity between T and G) allowed us to apply the T‐G continuum analysis, similar to above, to show a T‐to‐G transition both between visually‐ and motor‐responsive neurons and even within individual Visuomotor neurons similar to the memory‐guided gaze task (Figure 8). This time, however, the transformation occurred within the short interval of the response time (i.e. ~200 ms) and followed a similar progression in all neuron types. Thus, even in the absence of a memory period, as the activity evolved from visual‐to‐motor temporal codes, spatial representations evolved from an accurate target representation to one that closely reflects the inaccuracy in gaze endpoint. Overall, these studies suggest that the visuomotor transformation for gaze does not involve a discrete switch between target to gaze coding, but rather an intermediate progression that may or may not involve different neuron types, depending on timing and task details.

## 9 THEORETICAL IMPLICATIONS: A NEW CONCEPTUAL MODEL FOR GAZE CONTROL

**FIGURE 9:**
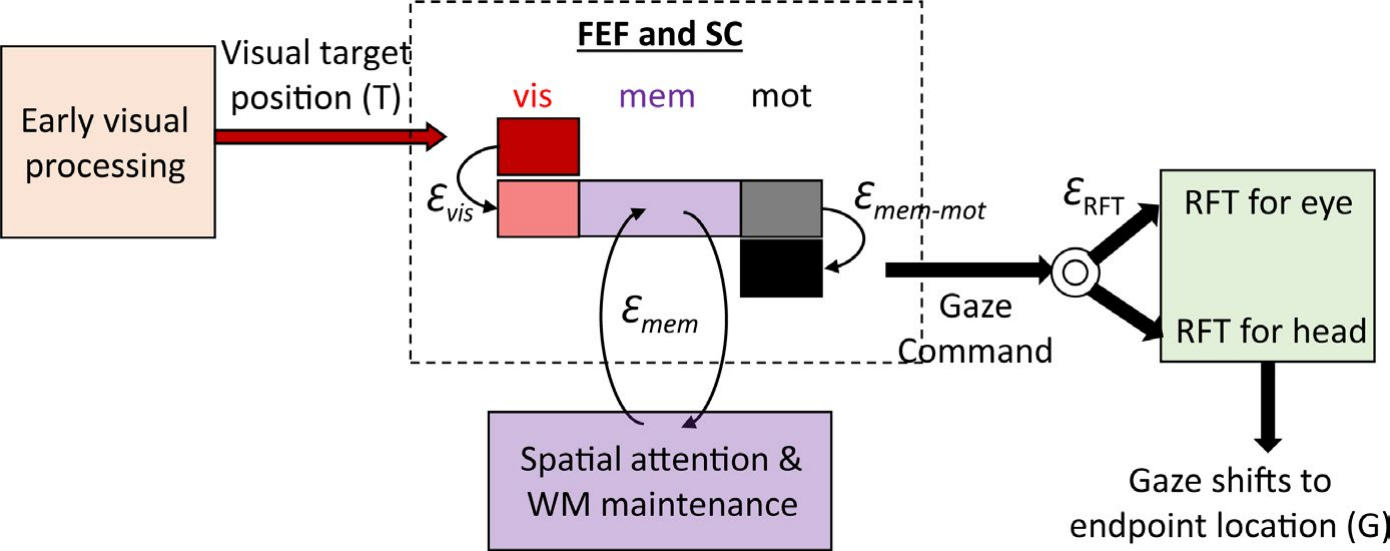
Conceptual model explaining visuomotor transformations in FEF and SC. A schematic of FEF and SC temporal responses during visual, memory, and motor periods (enclosed in dashed box) and relationship with different visuomotor processing stages are shown. Visual neurons (red) encoded the accurate target position in eye coordinates (T). These neurons receive projections from early visual processing areas. Visuomotor neurons (pink) encoded positions that fell close to T but drawn toward the direction that predicted gaze endpoint in eye‐centered coordinates (G). This visual response likely reflects a stage of visual processing which maps, through a noisy gate, visual information into a priority map of movement goals resulting in the accumulation of errors in behavior (*Ɛ*_vis_). This position is maintained through recurrent connections between frontal and parietal areas (purple box), which also send projections to FEF and SC. This memory maintenance is susceptible to noise (*Ɛ*_mem_), resulting in the diffusion of the attention spotlight (or memorized location). After the GO signal, the most recent memory of the target location is transferred, via a noisy output gate, to the motor circuitry, resulting in additional accumulation of noise (*Ɛ*_mem‐mot_). The motor neurons in FEF and SC send this gaze command to downstream structures, where additional processing for the coordination of effectors and appropriate reference frame transformations (RFT) take place. Adapted from Sajad et al., (2015, 2016)

The general conclusion of our FEF and SC findings seems clear: both structures shared very similar spatiotemporal progression of signals and transformations. This suggests extensive sharing of signals between the SC and FEF, likely through their interconnections (Munoz & Schall, 2004; Sommer & Wurtz, 2000, 2001, 2004). This further supports the notion that these two structures behave as a unit in the sensorimotor transformation for gaze shifts (or saccades in head‐restrained conditions), sharing both the desired transformation (designed to land gaze on target) and likely transformation‐related noise, resulting in the variable gaze errors that we measured and used in our analysis. Accordingly, these two structures are largely treated as one unit in the conceptual model that follows (Figure 9).

In order to construct the conceptual model illustrated in Figure 9, we synthesized our own results with knowledge derived from the previous literature. Our conceptual model relies on the assumption that visuomotor transformations are inherently noisy (Alikhanian et al., 2015; Arieli, Sterkin, Grinvald, & Aertsen, 1996; Churchland et al., 2006; Faisal et al., 2008; Gnadt et al., 1991; van Bergen et al., 2015; Wimmer et al., 2014).

The model begins with the frontal cortex and SC receiving the true location of the visual stimulus in eye‐centered (i.e. retinal) coordinates. This is based on the observation that visual response of Visual neurons in both FEF and SC was best described by the T model along the T‐G continuum (Sajad et al., 2016). Having access to this accurate visual information can be achieved by direct projections from visual cortex as well as the retina (May, 2006; Perry & Cowey, 1984; Schall et al., 1995).

To guide appropriate behavior, the information about target location needs to be gated to the appropriate memory and motor circuitry to meet the requirements of the task. Such a gating mechanism can be implemented by the corticocortical and cortico‐striato‐thalamic loops, and subcortical circuits through the basal ganglia (Battaglia‐Mayer & Caminiti, 2019; Coe et al., 2019; Krauzlis et al., 2013; Lynch & Tian, 2006; O'Reilly & Frank, 2006). These circuits can integrate various sensory information with learned associations to transfer the spotlight of attention onto relevant locations. One candidate for such a representation in FEF and SC can be the visual response of Visuomotor neurons. In our experiments, the visuospatial representation of Visuomotor neurons, at similar latency to that of T‐coding Visual neurons, was slightly shifted toward G, indicating accumulation of noise. This noise can arise due to the gating that transforms visual input into a movement goal (Figure 9, the noise is labeled *Ɛ*_vis_; Chatham & Badre, 2015; O'Reilly & Frank, 2006). Anatomically, this noise can arise from reduction in resolution due to synaptic integration in the basal ganglia circuits (Avery & Krichmar, 2015; Parthasarathy, Schall, & Graybiel, 1992; Zheng & Wilson, 2002). A leading hypothesis suggests that while this noise results in inaccurate behavior, it can offer the required flexibility to perform various cognitive transformations (Faisal et al., 2008; McDonnell & Ward, 2011). In complex tasks that involve stimulus‐response incompatibility (such as the antisaccade task), this noisy gate would transform visual information into a movement goal at a location indicated by the stimulus‐response mapping rule (Boettiger & D'Esposito, 2005; Dash, Yan, Wang, & Crawford, 2015; Everling & Johnston, 2013; Miller & Cohen, 2001; Munoz & Everling, 2004; Sato & Schall, 2003). In simple gaze tasks where the visual stimulus and movement goal are spatially congruent, it would simply transfer activity to the population of neurons that (roughly) represent the same patch of space (Marino et al., 2008; Spaak et al., 2017).

After the movement goal is determined, it needs to be maintained in working memory or directly routed to the motor network depending on the task requirements. It has been shown that the activity in FEF and SC during memory delay reflects the maintained representations in working memory (Funahashi, Bruce, & Goldman‐Rakic, 1989; Fuster & Alexander, 1971; Lundqvist, Herman, & Miller, 2018; Peel, Dash, Lomber, & Corneil, 2017; Sommer & Wurtz, 2001). We observed that there was a transition in spatial representations toward G during the memory delay period in both structures. This confirms models of spatial working memory that describe the diffusion of spatial representations in a random‐walk fashion due to accumulated noise in the population dynamics (Figure 9, label *Ɛ*_mem_; Compte, Brunel, Goldman‐Rakic, & Wang, 2000; Wimmer et al., 2014). Our finding that the memory responses did not exactly reach G suggests that this diffusion process does not fully account for gaze endpoint inaccuracy (Churchland et al., 2006; Faisal et al., 2008; Ma et al., 2014).

In our studies on memory‐guided gaze shifts, we only found a strong preference for G code (or very similar codes) in neurons that exclusively fired during the gaze shift, suggesting noise in a memory‐to‐motor transformation (Figure 9, this noise is labeled *Ɛ*_mem‐mot_; Ketcham, Hodgson, Kennard, & Stelmach, 2003; Ma et al., 2014; Ploner, Rivaud‐Péchoux, Gaymard, Agid, & Pierrot‐Deseilligny, 1999). We propose that this transformation involves a second noisy gating of maintained movement goal from visually and memory‐responsive neurons to purely motor‐responsive neurons with no memory activity (M‐only neurons), possibly involving striato‐thalamic circuits (Brown, Bullock, & Grossberg, 2004; Chatham & Badre, 2015; Schall et al., 2011). This is in agreement with previous studies that show differential contribution of distinct subpopulations to motor preparation and their differences in anatomical and functional connections (Basso & May, 2017; Cohen et al., 2009; Doubell, Skaliora, Baron, & King, 2003; Markowitz et al., 2015; Merrikhi et al., 2017; Ninomiya, Sawamura, Inoue, & Takada, 2012; Pouget et al., 2009; Ray, Pouget, & Schall, 2009; Redgrave et al., 2010; Segraves & Goldberg, 1987; Weyand & Gafka, 1998). Once the motor network,comprised of FEF and SC M‐only neurons, is triggered to threshold levels, a gaze command is sent to downstream motor structures (Klier et al., 2001; Sparks, 2002).

One might have noticed that in the overall motor response populations, the T‐G code did not quite made it all the way to G (Figures 6, 7, 8), leaving some error unaccounted for. This suggests additional noise in sensorimotor transformations downstream of the FEF and SC, as demonstrated previously (Figure 9; this noise is labeled *Ɛ*_RFT_; Alikhanian et al., 2015; Edelman & Goldberg, 2002; Frens & Van Opstal, 1997; Stanford & Sparks, 1994). We also found that the SC motor burst came closer to G in the memory‐guided task compared to reactive gaze shifts. (Sadeh et al., 2018). The most parsimonious explanation for this result is that the unaccounted downstream noise (i.e., *Ɛ*_RFT_) was equal in both cases, but would contribute proportionately less to the overall errors when additional memory‐related noise is present.

### 9.1 Future directions, emerging questions, and new hypotheses

The methodologies, results, and model described in this review can lead to many more questions, such as: (a) How is the T‐to‐G transformation accomplished through the interaction of neurons within and between different layers of SC and FEF microcircuits? (Basso & May, 2017; Bastos et al., 2012; Chandrasekaran, Peixoto, Newsome, & Shenoy, 2017; Heinzleet al., 2007; Massot, Jagadisan, & Gandhi, 2019; Sajad, Godlove, & Schall, 2019; Shin & Sommer, 2012); (b) How do the spatial codes at the individual neuron and population levels change in other visuomotor behaviors, such as express saccades (latency < 100 ms), in which the temporal visual and motor responses entirely overlap (Dorris, Pare, & Munoz, 1997; Isa, 2002)?; (c) how does this methodology extend to other areas of the brain involved in gaze control (Bremmer et al., 2016; Schneider, Dominguez‐Vargas, Gibson, Kagan, & Wilke, 2020)?

Further, the general applicability of the model‐fitting method described here provides the opportunity to investigate other models and other behaviors, so long as there is related spatially tuned activity in the brain and variations in the behavior to distinguish the models. For example, the current review only touches on egocentric models; we have already started applying these methods to investigate the neural coding of allocentric landmarks in the gaze system (Bharmauria, Sajad, Li, et al., 2020a; Bharmauria, Sajad, Yan, Sajad, Yan, Wang, & Crawford, 2020b). We have also started using this method to differentiate gaze, head, and reach transformations in frontal cortex during coordinated eye‐head‐hand reaches (Arora et al., 2019; Nacher et al., 2019). There is no reason to not take this further afield, such as the analysis of activity in areas involved in spatial navigation and spatial memory, including the hippocampus and entorhinal cortex, against ego‐ and allocentric models during complex tasks such as natural viewing and free‐moving navigation (Gulli et al., 2020; Meister & Buffalo, 2018).

Finally, since our T‐G continuum (or potential analogues like a T‐Hand continuum) provides a measure of neural contribution to behavioral noise, these methodologies are applicable to fitting pathological sensorimotor noise (Avery & Krichmar, 2015; Bays & Wolpert, 2007; Burns & Blohm, 2010). Errors in behavior have been commonly investigated to make inference about brain function in healthy and diseased populations. A growing trend in clinical studies is to compare systematic inaccuracies (such as amplitude gain) and (to a lesser extent) variable inaccuracies in movement in diseased populations to gain insight into the nature of their deficits (e.g. Ketcham et al., 2003; Ploner et al., 1999; Thakkar, Schall, Heckers, & Park, 2015). Our methods would allow one to trace this noise to specific neural transformations. For example, it could identify the source of noise for pathological saccades (Chan, Armstrong, Pari, Riopelle, & Munoz, 2005; Le Heron, MacAskill, & Anderson, 2005) or memory‐motor transformations in Parkinson's disease (Ketcham et al., 2003). Such tests are actually being done at this time.

## 10 GENERAL CONCLUSIONS

The visuomotor transformation for gaze control has been the subject of scientific investigation for decades. While this system is celebrated as a model for understanding general sensorimotor transformations and various cognitive functions, it is extraordinarily difficult to show how its spatial codes evolve through time. Some of this is due to complexity (e.g. accounting for the many possible models in head‐unrestrained behavior) and some ironically due to simplicity (i.e. due to the similarity of visual and motor vectors during ordinary saccades). But solving these technical problems has led us to a methodology with surprising power and versatility, including the ability to test simultaneously between all known models of this system, and track intermediate transformations (especially through the T‐G continuum) through time.

Having applied these methods to the SC and FEF during head‐unrestrained gaze shifts, with or without a memory delay, we find a similar preference for eye‐centered coding in both structures, with the visual response encoding T versus the motor response encoding positions closely described by G (i.e. future gaze position). In the studies reviewed here, we have found a progressive spatiotemporal transition through intermediate T‐G codes, with a memory delay, and a more rapid transition without a delay. This transformation was both local (occurring even *within* some neuron types) and global, appearing in parallel in these widely separated (but interconnected) brainstem and cortical structures. Importantly, this includes sharing of the neural noise that apparently both allowed us to distinguish T from G, and explains considerable behavioral errors. This does not mean that these structures do the same thing, but that the other functions they support are embedded within fundamentally similar sensorimotor “carrier waves” (Fuster, 2001; Wurtz et al., 2001). Finally, these conclusions likely generalize to other systems. For example, in the reach system, a transition from visual‐to‐motor coding has been observed both at the level of individual neurons, between neurons, and between areas in electrophysiological studies (Bremner & Andersen, 2014; Caminiti, Johnson, Galli, Ferraina, & Burnod, 1991; Cisek & Kalaska, 2005; Fujiwara, Lee, Ishikawa, Kakei, & Izawa, 2017; Kakei, Hoffman, & Strick, 2003; Pesaran, Nelson, & Andersen, 2006; Westendorff et al., 2010), and across lobes at the whole cortex level in neuroimaging studies (e.g. Blohm et al., 2019; Cappadocia, Monaco, Chen, Blohm, & Crawford, 2016; Gallivan & Culham, 2015). A general conclusion from this is that visuomotor transformations are not compartmentalized, but rather involve distributed signals that permeate and underlie many brain functions.

## CONFLICT OF INTEREST

The authors report no conflict of interest.

## AUTHOR CONTRIBUTION

AS and JDC wrote the manuscript. All authors contributed to the primary research (and figures) featured in this review.

## ETHICAL STATEMENT

The experiments conducted by the authors featured in this manuscript complied with the guidelines of Canadian Council on Animal Care on the use of laboratory animals and were approved by the York University Animal Care Committee.
